# Bioavailability and Bioactivity of Plant Antioxidants

**DOI:** 10.3390/antiox11122336

**Published:** 2022-11-25

**Authors:** Dorota Żyżelewicz, Joanna Oracz

**Affiliations:** Institute of Food Technology and Analysis, Faculty of Biotechnology and Food Sciences, Lodz University of Technology, 2/22 Stefanowskiego St., 90-537 Lodz, Poland

## 1. Introduction

Plant-derived antioxidants are a large group of chemical compounds that include the secondary metabolites of plants (e.g., polyphenols, carotenoids, tocopherols, and coumarins) and the substances formed in food during processing (e.g., Maillard reaction products). These compounds are widely distributed in fruits and vegetables, as well as their derived products. Natural antioxidants have been gaining increasing interest, mainly due to the association between their consumption and the prevention of cardiovascular disease, cancer, neurodegenerative disorders, diabetes, and some other illnesses. Many research studies on plant-derived antioxidants have shown that these compounds exhibit a wide range of biological effects, including anti-aging, anti-inflammatory, anti-atherosclerotic, and anti-cancer. The bioavailability and bioefficacy of plant-derived antioxidants, including polyphenols, carotenoids, and tocopherols, are dependent on their molecular structure, food matrix, the occurrence of other substances, and their digestion pathways. Recent evidence suggests that the pharmacokinetics and metabolism of bioactive compounds are crucial to understanding their role and function in human health. However, the exact mechanisms of action, effects, and bioavailability of plant antioxidants are still not fully recognized.

This Special Issue consists of 18 articles focusing on the field of fortified and functional foods, the impact of technological processes on the content and transformation of bioactive components and the antioxidant activity of food, as well as in vitro and in vivo studies of the bioavailability, bioaccessibility, and bioactivity of plant-derived antioxidants, and explaining the effect of metabolism and pharmacokinetics on the efficacy of plant antioxidants and other potentially health-promoting mechanisms.

## 2. Fortified and Functional Foods

The scientific literature provides many examples of the use of plant raw materials rich in antioxidants and by-products of their processing to obtain fortified or functional foods, especially products that are poor in antioxidants in traditional formulations. A rich source of many active substances with health-promoting properties is sea buckthorn. Among them, antioxidants, phytosterols, essential fatty acids, amino acids, and vitamins C, K, and E should be mentioned. Sea buckthorn shows antimicrobial and antiviral properties and is a potential nutraceutical or cosmeceutical. Moreover, it has been proven to help treat cardiovascular diseases, cancer, and diabetes, as well as gastrointestinal and skin problems. Sea buckthorn is the most often used in the production of foods such as juices, jams, wines, pies, and liquors. There are also known examples of the addition of this fruit or its preparations for the enrichment of frozen yoghurt, soy drinks fermented by *Lactobacillus casei*, and pork sausages [1]. Another example of raw material rich in bioactive ingredients with beneficial effects on human health, especially polyphenols, is chia seeds. Drużyńska et al. [2] determined the composition and content of selected biologically active compounds present in chia seeds, the possibility of enriching a food product with chia antioxidant compounds and changing the profile of these compounds by modifying the seeds through soaking, as well as the influence of the fermentation process on the content and the activity of these compounds in yoghurt. The authors also determined the antioxidant properties of chia seeds and yoghurts with the addition of these seeds and seeds soaked in apple juice using in vitro tests such as DPPH, ABTS, and the ability to chelate Fe(II) ions. The research results showed that yoghurts with added chia seeds show a good level of antioxidant properties, and the susceptibility of the seeds to the introduction of other ingredients by soaking allows for their targeted modification. The use of chia seeds as a source of important ingredients and as a carrier of additional compounds makes it possible to develop new functional products with a designed composition and activity [2]. Waste or by-products from the agro-food industry (i.e., apple or grape pomace) characterized by a high content of bioactive ingredients can also be used for food enrichment. Gumul et al. [3] used apple pomace to fortify gluten-free breads based on corn and potato starches. Among the received products, bread with 5% of apple pomace was selected as an innovative gluten-free product for people with gluten intolerance. This product showed the highest organoleptic scores and was characterized by 2.5 times more polyphenols, eight times more flavonoids, four times more chlorogenic acid, and 21 times more phloridzin content than the control, resulting in 6.5 times higher antioxidant potential [3].

Polyphenolic extracts, which could be used as functional food ingredients and whose qualitative–quantitative composition and properties depend on the extractant used, can be obtained from grape pomace. A study by Caponio et al. [4] showed that the hydroalcoholic extracts of grape pomace have greater antioxidant activity than aqueous extracts; this is due to the higher concentrations of anthocyanins, phenolic acids, flavonoids, and stilbenes. Research in the simulated in vitro gastric and intestinal digestion has demonstrated that the antioxidant activity of aqueous extracts increases after intestinal digestion, whereas the one relative to hydroalcoholic extracts dramatically decreases. In addition, water extracts inhibited the growth of the tested pathogens and promoted the growth of probiotic bacteria, despite their lower global antioxidant activity compared to hydroalcoholic extracts [4].

## 3. Bioactive Compounds in Technological Processes of Food Production

The cultivation conditions and the type and parameters of the production processes used affect the chemical composition and properties of raw materials as well as semi-finished and finished products. Research into the effect of UVB radiation on changes in the composition of primary and secondary metabolites in plants was conducted by Yeo et al. [5]. Purple kohlrabi sprouts (*Brassica oleracea* var. *gongylodes*) were used as research material. The study indicated that UVB irradiation could induce an overall alteration in metabolite composition. Increased levels of secondary metabolites, such as anthocyanins and phenolic acids and ABTS free radical scavenging ability were observed during UVB irradiation [5].

Thermal processes can cause changes in the chemical composition, structure of compounds, and the bioactive properties of food. For example, roasting affects the qualitative and quantitative composition of polyphenols, the formation of Maillard reaction products and, consequently, the antioxidant potential of carob kibbles [6]. Thermal treatment also causes the degradation of anthocyanins, flavonoids, and other polyphenols and reduces the antioxidant activity of the red grape skin extract to a degree depending on the process parameters used (time, temperature) [7]. Studies of the kinetics of the convective drying process, anthocyanin degradation, total phenolic content (TPC), and the antioxidant capacity (AC) of Peruvian blackberry bagasse, which is a natural resource of phytochemicals for the development of food additives or dietary supplement applications, showed that the drying process was characterized by a logarithmic mathematical model and the degradation of the initial anthocyanin content followed a first-order kinetic model. In addition, TPC and AC increased with drying time and temperature due to increased water evaporation [8].

## 4. Bioactivity and Bioavailability of Antioxidants

The bioavailability, bioaccessibility, and bioactivity and, consequently, pro-health potency of plant bioactive compounds depend on their molecular mass, chemical structures, concentration in food, as well as food matrix and digestion pathways. Studies provided by Fraisse’s team using a simulated gastrointestinal tract model on the bioavailability of cyanidin-3-*O*-glucoside (C3G) showed a low bioavailability of this compound and confirmed its significant instability under intestinal conditions [9]. The decomposition ratio of C3G was set at 70%. On the other hand, the maintenance of its antiglycooxidative abilities was observed. The increase in α-glucosidase inhibitory activity was evident even after the intestinal phase, suggesting that classical in vitro studies may underestimate C3G antidiabetic potential. The authors suggest that this may impact the regulating effects of postprandial hyperglycemia and its potential usefulness for diabetes management [9]. Other have described research that showed that red grape skin extract inhibited the activity of certain enzymes, such as α-amylase, α-glucosidase, lipase, and lipoxygenase, which are associated with metabolic syndrome (MS) (including with diabetes) and inflammation [7]. The analysis of the molecular models obtained in molecular docking studies showed that malvidin 3-*O*-glucoside binds in the vicinity of the catalytic site of α-amylase and lipase. In contrast, in the case of α-glucosidase and lipoxygenase, no direct contact with catalytic amino acids was found. However, further research involving biological assays is needed to confirm the possibility of using this extract as a natural agent to treat and reduce the incidence of MS [7].

A review article authored by Zugravu et al. [10] describes the main antioxidants of hop plants (*Humulus lupulus* L.), their bioavailability and their biological activity. It reports the most important results obtained so far in the primary and secondary prevention of several non-communicable diseases, such as cancer and MS, understood as a cluster of risk factors for diabetes mellitus and cardiovascular diseases (High-Density Lipoprotein cholesterol, triglycerides) in the presence of central obesity [10].

The bioavailability of phenolic compounds contained in food matrices can be enhanced by the prior application of various bioprocessing techniques. In the case of spelt seeds (*Triticum spelta* L. cv. *Ostro*), the highest relative bioavailability of p-coumaric acid and trans-ferulic acid showed seeds subjected to enzymatic treatment and then fermentation. These processes also allowed for the improvement of the antioxidant activity of the tested material [11]. Increasing the bioavailability and improving the bioactive properties of compounds known for their antioxidant activity and therapeutic benefits can be achieved by subjecting them to a glycosylation reaction. The research of Méndez-Líter’s team concerned the optimization of the production of three new epigallocatechin gallate (EGCG) glycosides (glucoside, sophorose, xyloside) by transglycosylation using two engineered glycoside hydrolases from the fungus *Talaromyces amestolkiae* [12]. The obtained derivatives differed in solubility (glucoside > xyloside > sophoroside) and thermal stability. In addition, glycosylation resulted in the improved bioactive properties of the derivatives compared to EGCG, manifested by better antiproliferative properties on breast adenocarcinoma cell line MDA-MB-231 than EGCG, and the glucosylated and sophorylated derivatives induced higher neuroprotection, increasing the viability of SH-S5Y5 neurons exposed to okadaic acid [12].

The study conducted by Rao et al. [13] allowed for the characterization of a wide range of amino acids and organic acids and their anti-radical activity in different sugarcane varieties and presented candidate genes that could be potentially valuable for the genetic improvement of metabolites in sugarcane bagasse. It includes the identification of 55 organic acid compounds and 72 amino acids present in the rinds of the six cultivated sugarcane varieties and the functional verification of related genes, which is an outstanding prelude to further research on metabolomics in sugarcane [13].

Andonova et al. [14] conducted research on the composition and concentration of phenolic compounds, antioxidant activity (tests: DPPH, ABTS, FRAP, CUPRAC), and the ability to protect DNA against the reactive oxygen species (ROS) of ethanol extracts from the aerial parts of the *Koelreuteria paniculata* tree, such as the flowers, the flower buds, leaves, and stem bark. Five flavonoids (rutin, hesperidin, quercetin, (-)-epicatechin and only in stem bark extract: (+)-catechin) and nine phenolic acids (rosmarinic, *p*-coumaric, salicylic, vanillic, protocatehuic, caffeic, syringic, only in stem bark extract: ferulic and only in leaf extract: gallic) were identified in the extracts). All of the tested extracts showed antioxidant and DNA-protective potential, most visible in the case of extracts from flower parts and leaves [14].

Carotenoids are colored compounds with lipophilic properties and poor solubility during digestion, resulting in low and variable bioavailability. On the other hand, they are characterized by high antioxidant activity affecting several health benefits. The effect of frequently consumed proteins (whey protein isolate, WPI; soy protein isolate, SPI; sodium caseinate, SC), gelatin, GEL; turkey and cod) on carotenoid bioavailability and cellular uptake was studied. The food content after joint digestion in the gastrointestinal tract of these proteins with food matrices rich in carotenoids (tomato and carrot juice and spinach) was studied in Caco-2 cell models. The matrices containing higher amounts of non-polar carotenes after protein addition were characterized by better fractional bioavailability during simulated digestion in the gastrointestinal tract than analogous systems of matrices rich in more polar xanthophylls. The digestion of matrices in the presence of protein promoted the cellular uptake of carotenes (β-carotene, up to 33%) and counteracted the negative effect of proteins on the bioaccessibility of xanthophylls by improving the cellular uptake of lutein + zeaxanthin (up to 12%), especially in the presence of WPI and SC proteins [15].

Flavanones are consumed in our diet almost exclusively with citrus fruits and, to a lesser extent, with some herbs or tomatoes. Hesperidin is the main flavanone present in oranges, and eriocitrin in lemons. The available scientific evidence shows that the intake of flavonoid-rich citrus fruits is associated with a lower risk of stroke and the occurrence of certain types of cancer. However, the low solubility of hesperidin hinders its bioavailability. A randomized human crossover pharmacokinetic study (*n* = 16) conducted by Ávila-Gálvez [16] compared the bioavailability and metabolism of flavanones from lemon and orange extracts, which showed that the ingestion of eriocitrin-rich lemon extract provides more and higher concentrations of circulating Phase-II flavanone metabolites than after the ingestion of extracts rich in hesperidin. The authors also suggest that the consumption of lemon extract may provide a sufficient threshold concentration of circulating metabolites to exert health benefits with long-term consumption [16].

The LC-ESI-LTQ-Orbitrap-MS was used to study the distribution of oleaceins (OLEA), oleocanthalu (OLC), and their metabolite derivatives in rat plasma and organs/tissues (brain, heart, kidney, liver, lung, small intestine, spleen, stomach, and skin) after the acute intake of refined olive oil. Unmetabolized OLEA was detected in the stomach, small intestine, liver, plasma and, above all, in the heart. OLEA metabolites, in turn, were mostly distributed in the liver, heart, spleen and lungs. OLC was detected only in gastrointestinal samples, while its metabolites were widely distributed in rat tissues, with the small intestine and liver being the most important metabolizing organs. The obtained results may partially explain the mechanism of the beneficial effect of extra virgin olive oil consumption. However, further detailed pharmacodynamic studies are needed to link the ingested concentrations of OLEA and OLC and the composition of their metabolites with the frequency of the intake and the effect on the human body [17,18].
